# Appendix of Tables of French and English Weights and Measures

**Published:** 1828-05

**Authors:** 


The translator, Dr. Atlee, has not substituted, as we think he ought, the English for the French weights and measures, throughout the body of the work; but given an appendix of comparative tables. For the use of those who may not understand the subject, we shall subjoin them.

### TABLES,

GIVING A COMPARATIVE VIEW OF THE FRENCH WEIGHTS AND MEASURES WITH THOSE USED IN THE UNITED STATES.

| *French* grains. | *English* grains. | *French* grains. | *English* grains. |
|---|---|---|---|
| 1 | 0,8203 | 6 | 4,9223 |
| 2 | 1,6407 | 7 | 5,7427 |
| 3 | 2,4611 | 8 | 6,5631 |
| 4 | 3,2815 | 9 | 7,3835 |
| 5 | 4,1019 | 10 | 8,2030 |

| *grain* | | | | *English grs.* | *Troy weight.* |
|---|---|---|---|---|---|
| 1 | | | . . . . . . = | 0,8204 | |
| 72 | *gros* | | . . . . = | 59,0703 or | . . ℈ij. 19,0703 grs. |
| 576 | 8 | *ounce* | . = | 472,5625 or | . ʒvij. 52,56 " |
| 9216 | 128 | 16 | 1 *pound*= | 7561,0000 or | ℥xv. ʒvj. 1, " |

The above French division is that called *pois de marc*, being that formerly used in France. It is this measure that is used in the body of this work. The English division to which it is reduced is that of *troy weight.*

I have given below the new French divisions, reduced to troy weight also.

| *French division.* | | *Troy weight.* grains. | pd. | oz. | dr. | sc. | gr. | |
|---|---|---|---|---|---|---|---|---|
| Milligramme | = | ,0154 | | | | | | |
| Centigramme | = | ,1544 | | | | | | |
| Decigramme | = | 1,5444 | | | | | | |
| Gramme | = | 15,4440 | | | | | | |
| Decagramme | = | 154,440 | 0 | 0 | 2 | 1 | 14 | 44-100 |
| Hecatogramme | = | 1544,400 | 0 | 3 | 1 | 2 | 4 | 4-10 |
| Kilogramme | = | 15444,023 | 2 | 8 | 1 | 0 | 14 | |

The French litre contains 2,1133 English pints, and the French pint 2,0171 English; so that the difference between them and the English quart measure is but trifling, and they may be used as synonymous. The same may be said of their gros and our drachm.

| *Troy weight.* *grains.* | | | | | | *French grains.* | |
|---|---|---|---|---|---|---|---|
| 1 | | | | | = | 1,218 | |
| 20 | *scruple* | | | | = | 24,378 | |
| 60 | 3 | *drachm* | | | = | 73,135 | 1 gros. 1,135 gr. |
| 480 | 24 | 8 | *ounce* | | = | 585,083 | 1 oz. 9,083 gr. |
| 5760 | 288 | 96 | 12 | 1 *pound* | = | 7021, | 12 oz. 1 gros. 27 gr. |

